# Household crowding as a potential mediator of socioeconomic determinants of tuberculosis incidence in Brazil

**DOI:** 10.1371/journal.pone.0176116

**Published:** 2017-04-18

**Authors:** Daniele Maria Pelissari, Fredi Alexander Diaz-Quijano

**Affiliations:** 1 National Tuberculosis Program of Brazil, Ministry of Health, Brasília, Distrito Federal, Brazil; 2 Department of Epidemiology, School of Public Health, University of São Paulo, São Paulo, São Paulo, Brazil; Chinese Academy of Medical Sciences and Peking Union Medical College, CHINA

## Abstract

Although many studies have identified social conditions associated with tuberculosis, contextual and individual factors have rarely been analysed simultaneously. Consequently, we aimed to identify contextual and individual factors associated with tuberculosis incidence in general population in Brazil in 2010. We also assessed whether household crowding mediates the association between socioeconomic determinants and tuberculosis incidence. Individual data of tuberculosis cases were obtained from 5,565 municipalities in Brazil in 2010 (last year of national census), and merged with contextual variables. The associations were evaluated in a multilevel analysis using negative binomial regression. After adjusting for individual factors (age, sex and race) and geographic region, the following contextual factors were associated with tuberculosis incidence rate: AIDS incidence rate [incidence rate ratio (IRR), 1.21; 95% confidence interval (CI), 1.18–1.24], unemployment rate (IRR, 1.16; 95% CI, 1.13–1.19), Gini coefficient (IRR, 1.05; 95% CI, 1.02–1.08), proportion of inmates (IRR, 1.11; 95% CI, 1.09–1.14), mean per capita household income (IRR, 0.94; 95% CI, 0.91–0.97) and primary care coverage (IRR, 0.94; 95% CI, 0.92–0.96). Inclusion of household crowding in the multivariate model led to a loss of the associations of both Gini coefficient and mean per capita household income. In conclusion, our findings suggest that income inequality and poverty, as determinants of tuberculosis incidence, can be mediated by household crowding. Moreover, prison population can represent a potential social reservoir of tuberculosis in Brazil and should be addressed as a priority for disease control. Finally, the negative association between primary health coverage and tuberculosis incidence highlights the importance of this level of care as a strategy to control this disease.

## Introduction

Tuberculosis control still poses a challenge to public health services worldwide. In 2015, there were an estimated 10.4 million cases, primarily in Asia (61%) and Africa (26%). The America Regions contributed with 268,000 cases (3%), and Brazil had approximately 84,000 cases, ranking 20th among the 30 countries with the highest burden in terms of absolute numbers of tuberculosis incident cases [1].

Several factors have been associated with tuberculosis, including endogenous factors (age, sex, race, the presence of HIV and diabetes) and lifestyle habits (alcohol consumption) [2–4]. This approach, though it identifies risk factors at the individual level, does not consider the socio-economic context of the population, which is a major determinant of the tuberculosis incidence [5].

At the community level, previous research has identified the contribution of macro-determinants to tuberculosis occurrence, such as income inequality [6,7], poverty [6,8], gross domestic product (GDP) per capita [9,10], population density [8,11], household overcrowding [12–15], low educational level of the population [12,14–17], unemployment rate [12,14–19] and epidemiological phenomena, such as the incidence of Acquired Immune Deficiency Syndrome (AIDS) [10,20,21].

Regarding socioeconomic determinants, a high proportion of low and middle country residents live in destitution conditions characterized by household crowding [22,23] which may directly favours tuberculosis transmission [12–15]. In addition, the association between tuberculosis and imprisonment, and the effect that the proportion of inmates has on tuberculosis incidence in general population are already recognized [24]. However, the effect of all of these variables on tuberculosis incidence has not been simultaneously evaluated in multiple models considering both individual and contextual levels.

In Brazil, the system for tuberculosis surveillance has national coverage and mandatory reporting. Additionally, the socioeconomic data of 5,565 Brazilian municipalities are available through the latest demographic census [25]. The availability of such data provides an opportunity to compare different economic situations that may be associated with the incidence of tuberculosis.

Consequently, we performed a multilevel analysis to identify macro-determinants independently associated with tuberculosis incidence rate in general population in Brazil in 2010. Moreover, we assessed whether household crowding mediates the association between socioeconomic determinants and tuberculosis incidence.

## Methods

### Study design and data sources

This is an ecological population-based study that was conducted using data from Brazil on tuberculosis incidence and population macro-determinants. Data were available for 2010, the last year of the national census, when Brazil registered 190,732,694 inhabitants distributed in 5,565 municipalities [25].

The individual characteristics of tuberculosis patients (all clinical forms) diagnosed in 2010 were obtained from the Brazilian National Information System for Notifiable Diseases (Sistema de Informação de Agravos de Notificação [SINAN]) [26]. The socioeconomic factors of the municipalities were obtained from the census [25] and the Human Development Atlas [27]. The proportion of inmates by municipality was estimated from the National Survey of Penitentiary Information [28].

Ethical approval was granted by the School of Public Health Research Ethics Committees from University of São Paulo (number: 1.553.841).

### Variables

The individual variables analysed were: sex (women and men), age (0–14, 15–59 and 60 years and older) and race black (black and brown) and non-black (white, yellow and indigenous). For contextual variables, we analysed the following socioeconomic indicators: human development index, mean per capita household income, GDP per capita, proportion of extremely poor/poor/vulnerable to poverty, Gini coefficient, unemployment rate, illiteracy, population density (people per km^2^) and the proportion of inmates. We based in the Human Development Atlas to define household crowding as the proportion of the population living in households with more than two people per bedroom [27]. In order to consider geographic differences, we also evaluated the region in which municipality is located (North, Northeast, Midwest, Southeast or South).

As measures of the quality and coverage of health services, we analysed respectively the infant mortality rate per 1,000 live births [9,10] and the primary care coverage, since the majority of tuberculosis cases in Brazil are diagnosed and followed-up in this level of care [29]. We also considered the AIDS incidence rate per 100,000 people and life expectancy at birth.

The values of the variables GDP per capita and mean per capita household income were converted from the Brazilian currency (reais- R$) to US dollars (USD), according to the average price in 2010, when USD$1.00 was worth R$1.76.

The proportion of extremely poor people was defined as the proportion of individuals with a per capita household income equal to or less than USD$40 (R$70), and poor people were defined as individuals with a per capita household income equal to or less than USD$80 (R$140). The cut-off income values for the vulnerable to poverty group was USD$ 145 (R$255), which corresponds to the half of the Brazilian minimum salary in 2010 (USD$290 or R$510) [27].

The AIDS incidence rate was calculated with data from SINAN, adopting Caracas’s criteria as the case definition, which has been used since the 1990s in Brazil [30].

The number of inmates at the municipal level was available only for 2014, whereas for the remaining years the information refers to the states. From 2010 to 2014 in Brazil, this population increased by 18% from 496,323 to 588,745; the increase was heterogeneous between the states [28]. To estimate the proportion of inmates for each municipality for 2010, we projected the inmate populations based on state variation, keeping the proportions that were observed in the municipalities in 2014.

### Statistical analysis

The mean, standard deviation, median, minimum and maximum values were calculated for the independent variables. The quantitative variables were standardized by subtracting the mean and dividing by the standard deviation (SD) ([X—mean]/SD), which allowed the generation of comparable measures of association in terms of the standard deviations for each independent variable.

In an initial analysis using a Poisson regression, overdispersion of the data was found. Because there were variables from both the individual level of population groups and the contextual level of municipalities, we opted to use a multilevel analysis with negative binomial regression, which has also been used in similar ecological studies on tuberculosis incidence [13–15,19]. Thus, this analysis considered two levels: an individual level including only demographic variables (sex, age and race); and a contextual level considering all other variables characterizing the municipalities.

Initially, each of contextual variables was evaluated in independent regression models adjusted only for the demographic variables (sex, age and race). Those contextual variables that presented *P* < 0.10 were analysed in the correlation matrix. To avoid collinearity among those with higher correlations (more than 0.60), we selected the variables with the highest associations with tuberculosis incidence.

Multiple analyses were performed starting with a model of demographic variables (in the individual level). Then contextual variables were added progressively considering the strength of the association with tuberculosis incidence. Variables with a *P* < 0.05 were considered statistically associated with the incidence and were preserved in the model. Region was retained in the model as a potential confounder.

We used the Sobel test to assess the mediation effect of household crowding on the association between socioeconomic variables and the tuberculosis incidence rate. In this paper, we considered a mediator as a variable that occurs in a causal pathway from an independent to a dependent variable. It causes variation in the dependent variable, and itself varies by the independent variable [31].

In the multilevel analysis, household crowding was only included in the final model to test its potential role as a mediator of the association of socioeconomic macro-determinants with the tuberculosis incidence. Operationally, when a mediator is included in a regression analysis model with the independent variable, the effect of the independent variable is reduced and the effect of the mediator remains significant. All of the analyses were performed using Stata (Version 11, 2009; College Station, TX).

## Results

**Fig 1:**
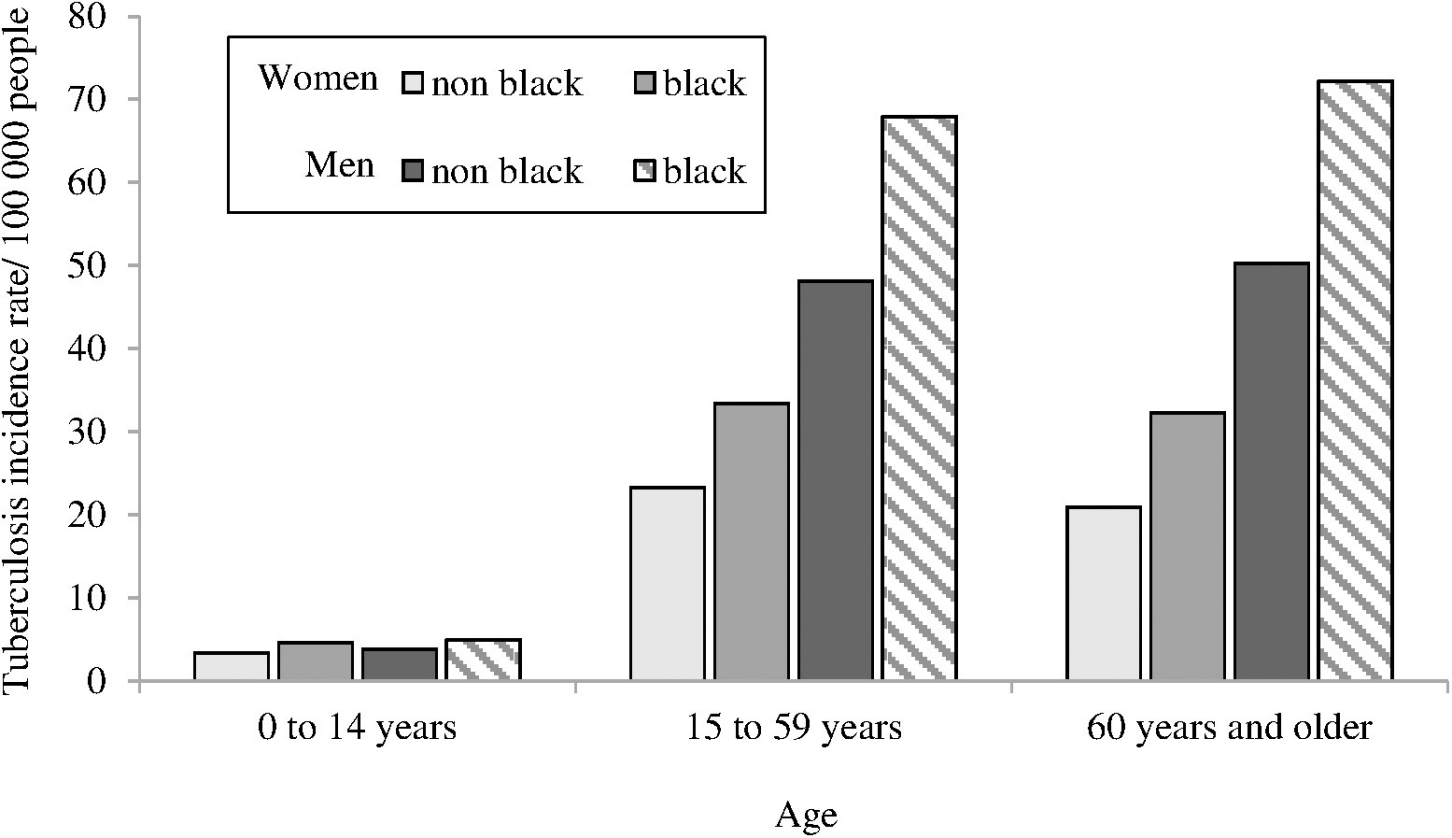
Tuberculosis incidence rate by age, sex and race. Brazil, 2010.

There were 71,610 new tuberculosis cases reported in 2010, resulting in an incidence rate of 37.5 per 100,000 people. A total of 63,907 (89.3%) cases, distributed in 3,839 municipalities (69%), had information about age, sex and race and were included in this study. The tuberculosis incidence rate was higher in men than in women, except for people younger than 15 years old. Black people, regardless of sex and age, had a higher risk for tuberculosis compared to nonblack people (Fig 1).

The Gini coefficient in 2010 in Brazil was 50.3%. However, there were municipalities with good income equality (e.g., São José do Hortência, 28.4%) in the South region and municipalities with extreme income inequality (e.g., São Gabriel da Cachoeira, 80.8%) in the North region. This pattern of high variability was also observed for other variables such as human development index, mean per capita household income and GDP per capita (Table 1).

**Table 1:** Description of contextual variables and estimations of its associations with tuberculosis incidence, using multilevel negative binomial regression. Brazil, 2010.

Variable	Mean (SD)	Median (min-max)	Adjusted IRR^a^	95% CI
Human development index	0.659 (0.072)	0.665 (0.418–0.862)	0.99	0.97–1.02
Mean per capita household income (U$)	274.7 (135.7)	259.3 (54.3–1,141.5)	0.97	0.95–0.99
GDP per capita (U$)	7,264.7 (8,035.4)	5,575.2 (1,291.2–168,628.6)	1	0.98–1.02
% extremely poor	11.5 (11.8)	6.6 (0.0–69.7)	1.02	0.99–1.05
% poor	23.2 (17.9)	18.2 (0.2–78.6)	1.03	1.01–1.06
% vulnerable to poverty	44.0 (22.4)	42.2 (2.0–91.6)	1.04	1.01–1.06
Gini coefficient (%)	50.3 (6.6)	50.3 (28.4–80.8)	1.09	1.06–1.11
Unemployment rate	6.3 (3.7)	5.8 (0.1–39.2)	1.18	1.16–1.21
Illiteracy rate of those 18 years and older	17.4 (10.7)	14.1 (1.0–47.6)	0.98	0.95–1.00
Illiteracy rate of those 15 years and older	15.8 (9.8)	12.9 (0.9–47.1)	0.98	0.95–1.00
Household crowding	25.1 (13.0)	23.1 (0.7–88.6)	1.2	1.17–1.23
Population density (inh./km^2^)	108.2 (572.4)	24.4 (0.13–13 024.6)	1.03	1.01–1.05
% inmates	0.2 (1.7)	0.0 (0.0–52.8)	1.13	1.11–1.15
AIDS incidence rate/100,000 inh.	9.1 (13.9)	2.8 (0.0–224.7)	1.22	1.19–1.25
Infant mortality rate/1,000 live births	19.2 (7.1)	16.9 (8.5–46.8)	1.02	0.99–1.04
Life expectancy at birth	73.1 (2.7)	73.5 (65.3–78.6)	0.97	0.96–1.01
Primary care coverage	87.4 (20.6)	100 (0–100)	0.9	0.88–0.92
**Case distribution by region—N° (%)**				
North	6,897 (10.8)		Reference	
Northeast	17,427 (27.3)		0.83	0.76–0.90
Midwest	2,973 (4.7)		0.74	0.66–0.83
Southeast	27,980 (43.8)		0.74	0.68–0.81
South	8,630 (13.5)		0.79	0.72–0.87

GDP, gross domestic product; AIDS, Acquired Immune Deficiency Syndrome; SD, standard deviation; IRR, incidence rate ratio; CI, confidence interval.

^a^ Adjusted only by variables of the individual level: age, sex and race.

The following contextual variables, adjusted for sex, age and race, were associated with the tuberculosis incidence rate: mean per capita household income, proportion of poor and vulnerable to poverty, Gini coefficient, unemployment rate, household crowding, population density, proportion of inmates, AIDS incidence rate, primary care coverage and geographic region (Table 1).

**Fig 2:**
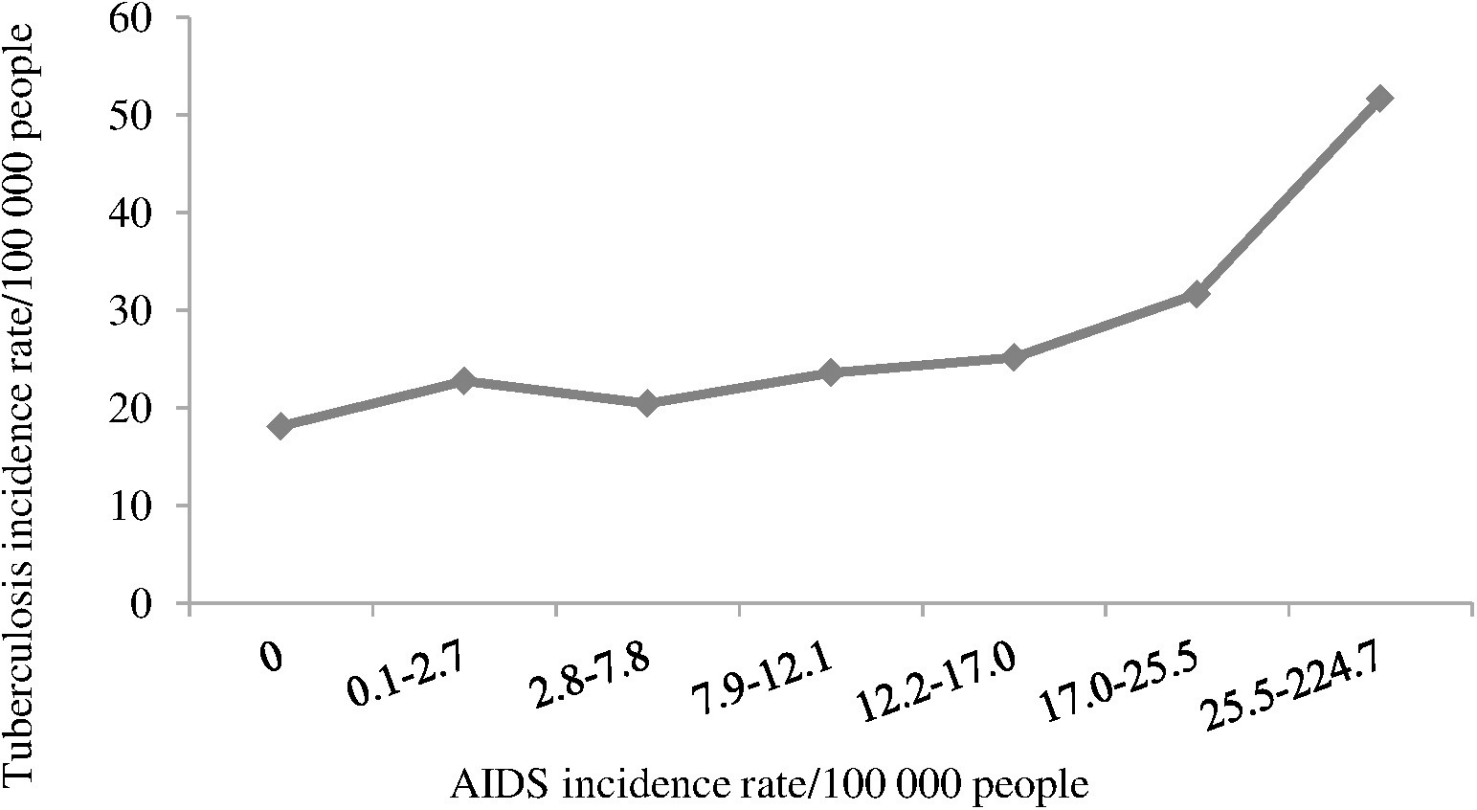
Tuberculosis incidence rate by AIDS incidence rate. Brazil, 2010.

Among the contextual variables, we highlighted the AIDS incidence rate because every 13.9 AIDS cases per 100,000 people represented a 22% increase in tuberculosis risk (Table 1). This increase is more evident when AIDS incidence rate was higher than 17 cases per 100,000 people (Fig 2).

The multilevel model, including all contextual variables selected (except household crowding), indicates consistent associations between the individual level variables and the tuberculosis incidence rate (Table 2). Compared to the population younger than 15 years old, those 15–59 years old [incidence rate ratio (IRR), 10.02; 95% confidence interval (CI), 9.51–10.56] and 60 years old (IRR, 10.68; 95% CI, 10.09–11.31) had a higher risk. Men (IRR, 2.07; 95% CI, 2.03–2.11) and black people (IRR, 1.41; 95% CI, 1.38–1.44) also had a higher risk of tuberculosis.

**Table 2:** Multilevel model with negative binomial regression on the association among the tuberculosis incidence rate and the independent variables. Brazil, 2010.

Variables		IRR^a^	95% CI
**Individual level**			
** **	Age		
** **	< 15 years	Reference	
** **	15–59 years	10.02	9.51–10.56
** **	60 years and older	10.68	10.09–11.31
** **	Sex		
** **	Women	Reference	
** **	Men	2.07	2.03–2.11
** **	Race		
** **	Non Black	Reference	
** **	Black	1.41	1.38–1.44
**Municipality level**			
** **	AIDS incidence rate/100,000 people	1.21	1.18–1.24
** **	Unemployed rate	1.16	1.13–1.19
** **	% of inmates	1.11	1.09–1.14
	Gini coefficient	1.05	1.02–1.08
	Household income (mean per capita)	0.94	0.91–0.97
	Primary care coverage		0.94	0.92–0.96
	Region		
** **	North	Reference	
	Northeast	0.89	0.82–0.97
	Midwest	0.89	0.79–1
	Southeast	0.88	0.79–0.97
	South	1.02	0.91–1.15

AIDS, Acquired Immune Deficiency Syndrome; IRR, incidence rate ratio; CI, confidence interval.

^a^The association measure represents the incidence rate ratio for each standard deviation of the independent variable.

In this model, the contextual indicators positively associated with the tuberculosis incidence rate were the AIDS incidence rate, the unemployment rate, the Gini coefficient and the proportion of inmates (Table 2). Each increase of one standard deviation (SD = 13.9) in the AIDS incidence rate represented a 21% increase in the tuberculosis risk (IRR, 1.21; 95% CI, 1.18–1.24). For the unemployment rate (SD = 3.7), Gini coefficient (SD = 6.6) and proportion of inmates (SD = 1.7), each standard deviation led to an increase of 16%, 5% and 11%, respectively, in the tuberculosis incidence rate. Additionally, tuberculosis incidence was inversely associated with both the mean per capita household income (SD = 135.7) and primary health coverage (SD = 20.6). For those two variables, each standard deviation was associated with a 6% decrease in tuberculosis incidence (Table 2). Regarding geographic location, Northeast and Southeast regions exhibited lower tuberculosis incidence compared with North region (Table 2).

### Evaluation of household crowding as a mediator of socioeconomic determinants

**Fig 3:**
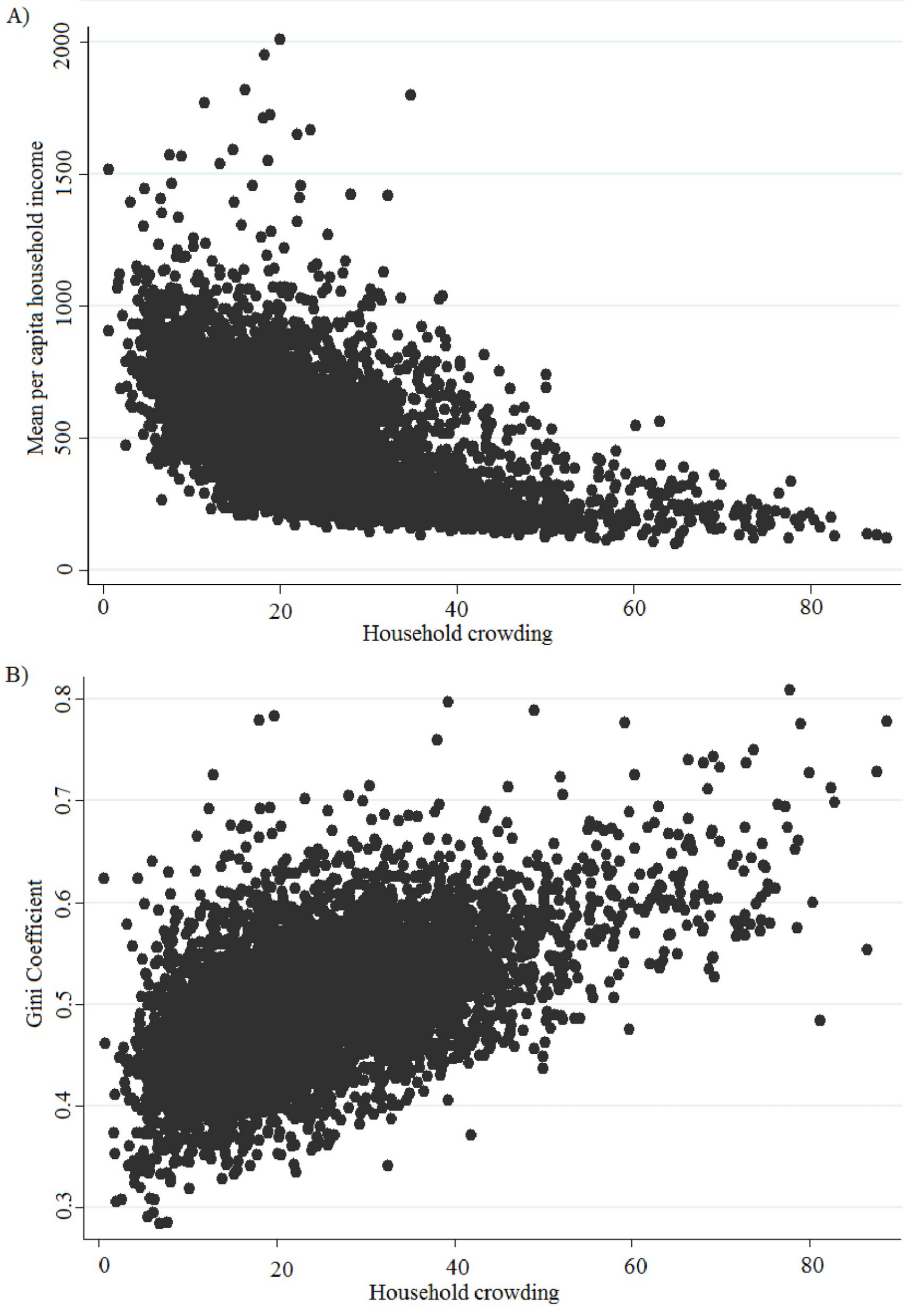
Correlation between household crowding and socioeconomic variables: Gini coefficient and mean per capita household income.

Household crowding was strongly correlated with both the mean per capita household income (Spearman = -0.68; p<0.001) and Gini coefficient (Spearman = 0.53; p<0.001) (Fig 3). Furthermore, the Sobel tests demonstrated significant mediation effect of household crowding on the associations between both of these variables and the tuberculosis incidence rate (p<0,001).

**Fig 4:**
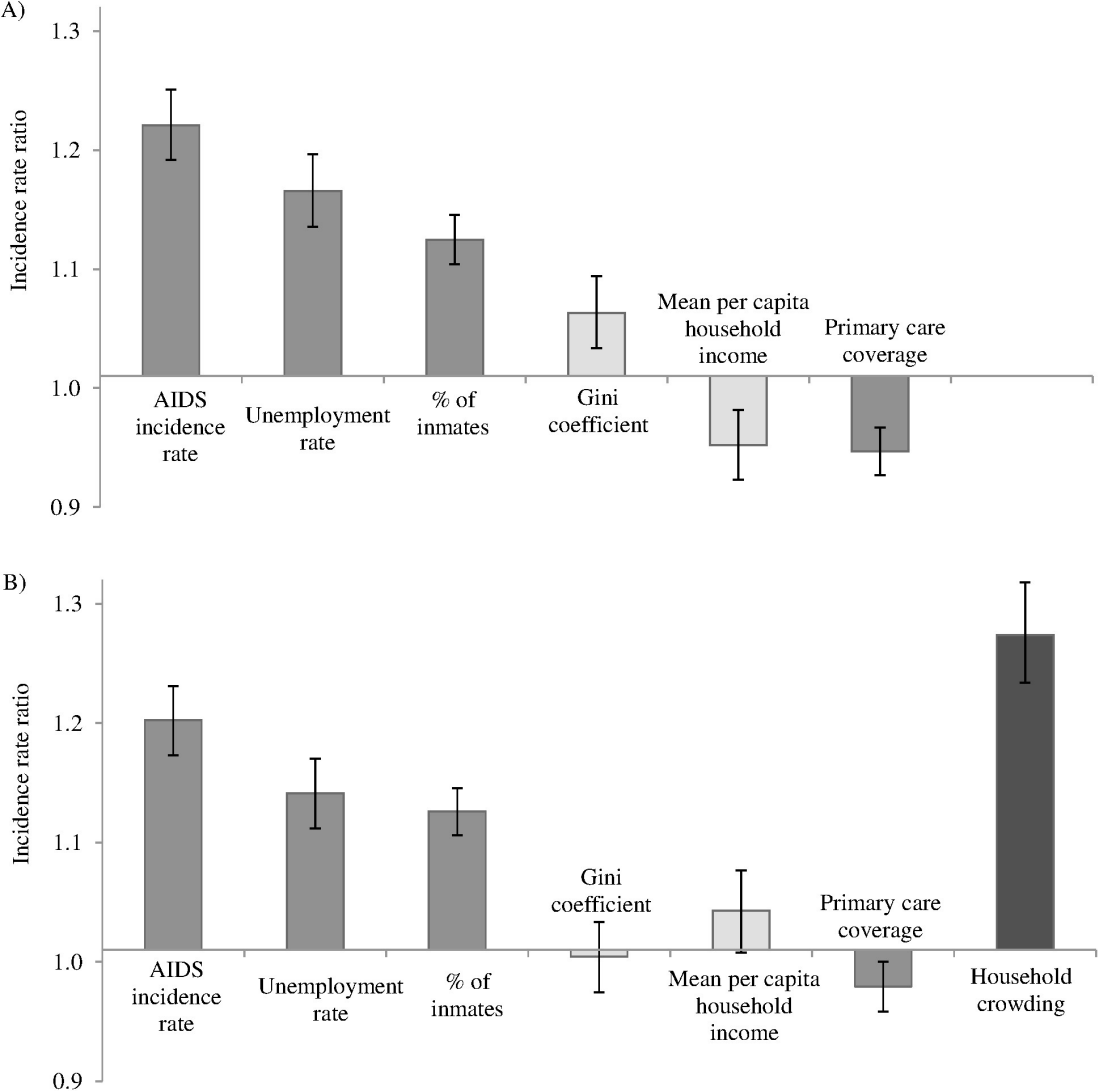
Effect of household crowding on estimations for socio-economic factors associated with tuberculosis. The incidence rate ratio reflects the change that occurs in the incidence rate when the independent variable increases by one standard deviation. Both models (A and B) are adjusted for region and the individual level variables (sex, age and race).

In the multilevel analysis, the inclusion of household crowding in the final model resulted in a loss of association of both Gini coefficient and mean per capita household income with tuberculosis incidence (IRR, 0.99; 95% CI = 0.97–1.02 and IRR, 1.03; 95% CI, 1.00–1.07, respectively). However, the association between household crowding and tuberculosis incidence remains significant (IRR, 1.26; 95% CI, 1.22–1.31) independently of the contextual and individual factors. No other association was ostensibly modified after household crowding was included in the final model (Fig 4).

## Discussion

This study, which has national coverage with a large population and a large variety of contexts, identified the importance of both socio-economic contextual and biological factors (AIDS incidence rate) in the tuberculosis incidence, after adjusting for geographic region and individual factors (sex, age and race). Moreover, tuberculosis incidence was associated with other contextual variables such as primary care coverage and the prevalence of an important vulnerable population for tuberculosis: inmates.

As in previous studies [10,20,21] the AIDS incidence rate was directly associated with the occurrence of new tuberculosis cases. In our study, this association remained strong even with the inclusion of other explanatory variables. The syndemic TB/HIV context is a special challenge for tuberculosis control because these two diseases interact synergistically, thereby amplifying the magnitude of the burden of disease [32].

The unemployment rate was also positively associated with the risk of tuberculosis. This result is consistent with those in high-income countries such as the United States (IRR, 1.2; P < 0.001) [18], Spain (adjusted relative risk, 1.18; 95% CI, 1.1–1.3) [16] and also in a previous local study from Brazil [odds ratio (OR), 1.31; 95% CI, 1.13–1.52] [20], suggesting that the association with this factor might be independent of the country's development.

Our results support a positive association between the tuberculosis incidence rate and the income inequality distribution as well as an inverse association of this disease with the mean per capita household income. These results are consistent with trends observed in other countries. For example, doubling the GDP was associated with a 38.5% decrease in the incidence of tuberculosis in 171 World Health Organization member countries [9]. Moreover, heterogeneity in the distribution of tuberculosis cases was explained by demographics and low household income in China [8]. Previous local studies also reported an association between tuberculosis incidence and low income in some regions from Brazil [6,20].

The positive association between household crowding, measured as the proportion of the population with more than two people per bedroom, and tuberculosis incidence is consistent with previous studies. Population density has been associated with the incidence of tuberculosis in developed countries, such as Italy (*P* = 0.002) [12], and the United States (household with more than one occupant per room rate ratio, 1.6; 95% CI, 1.5–1.6) [15]. Also in the West Africa, people living in houses with 6 to 10 adults (OR, 1.40; 95% IC, 1.05–1.85) and more than 10 adults (OR, 2.67; 95% IC, 1.64–4.34) had increased risk to tuberculosis compared with those living with less than 5 people [14].

Poverty and income inequality may be determinants of tuberculosis risk through different mechanisms, including individual [2–4] and contextual factors. However, we hypothesized that household crowding may act as a key mediator between socioeconomic determinants and tuberculosis incidence. In that sense, our results evidenced a strong relationship between household crowing and the indicators of poverty and inequality. This is consistent with previous studies suggesting that a crowded environment is usually a consequence of poverty [22,23]. On the other hand, household crowding may directly favours tuberculosis transmission by increasing the contact rate between *Mycobacterium tuberculosis* and susceptible people [14,23,33]. Therefore, crowding can be in the pathway relating socioeconomic determinant and risk of tuberculosis. This statement is consistent with results of the multilevel analysis because household crowding seems to explain the all effect that the income and inequity variables have on the tuberculosis incidence. This could suggest that overcrowding problems would be a specific target of intervention to mitigate the effect of poverty and inequity on the tuberculosis risk.

This study also evaluated the contribution of the proportion of inmates on the tuberculosis incidence adjusted for several contextual socioeconomics variables and individual demographic determinants. In prisons, the risk for tuberculosis is higher as a result of poor environmental conditions, overcrowding and poor access to health services [24]. The incarcerate rate increasing, which is a phenomenon recently observed in Brazil [28], is a challenge for the control of the disease. Although prisons are closed institutions, *M*. *tuberculosis* finds opportunities for transmission through the flow of people. As a consequence, this epidemiological observation highlights the interdependence of illness risks both inside and outside prisons. Our findings suggest that prisons play an important role in the tuberculosis burden in Brazil and represent a potential social reservoir of disease.

Previous studies suggest that the expansion of health primary care coverage in Brazil since 90^th^ was responsible for the improvement of health indicators [34,35]. Consistently, this study evidenced that primary health coverage was negatively associated with tuberculosis incidence suggesting a protective effect. A possible explanation for this result is that early diagnosis and appropriate follow-up in those services contribute with disease control by interrupting chain transmission.

### Limitations

Inferences obtained in the present study are essentially applicable to population groups because aggregated measures may differ from individual characteristics [36]. Therefore, its extrapolation to an individual level should be done cautiously and based on complementary evidence. For example, in the absence of an income variable of the tuberculosis cases, it was not possible to analyse this factor at the individual level. However, the inclusion of demographic factors could be considered an indirect assessment of the individual socioeconomic risk factor for tuberculosis. Even so, it is recommendable to develop specific analyses in regional and local scenarios in order to support focused interventions.

Although Brazil is consistently increasing case detection and notification [1], underreporting of tuberculosis is a potential limitation. We hypothesise that underreporting may be either non-differential among municipalities or more frequent in low-income municipalities. In both situations, the actual magnitude of the association between socioeconomic factors and tuberculosis incidence would be higher than that observed in this study.

The natural history of tuberculosis is a complex process that arises from different factors, whether individual or contextual. Although our model includes many of these factors, other important variables were not available for this analysis, such as malnutrition, diabetes, alcohol and tobacco consumption [2,4].

Despite these limitations, our model was based on population data with national coverage from an information system that detects at least 87% of the tuberculosis cases in a country of continental dimension [1]. Our sample of 5,565 municipalities allowed us to evaluate individual and contextual factors simultaneously. Moreover, the high variability observed among the analysis units allows the extrapolation of our results to other locations. In addition, ecological studies provide an overview that contributes in setting priorities and decision-making in public policy.

### Public health implications

A large decrease in the tuberculosis incidence occurred during the 20th century in high-income countries, long before the development of vaccines, diagnostic tests and antibiotics; this decrease occurred as a result of improving socioeconomic indicators in populations [10]. In this context, the “End TB Strategy” adopted in May 2014 by the World Health Assembly proposes to end the disease by 2035. This strategy suggests that actions must focus not only on the health sector response but also on the environment and the socioeconomic conditions of vulnerable populations [37].

More emphasis should be placed on interventions to reduce the susceptibility of tuberculosis infection and progression to active disease. Without the proper planning of urban space, infrastructure investments in housing conditions and policies that balance socioeconomic inequalities, poverty and income inequality will continue to play an important role in the incidence of tuberculosis. Interventions such as social protection policies, as well as, strengthening of primary health care, which also affect the prevention of tuberculosis [38], can be important tools for the disease control.

Additionally, the role of inmates as a contributor in the tuberculosis incidence at the municipality level suggests that incarceration is an important social reservoir of the disease, thus highlighting the necessity for prioritization of this population. Future studies should consider this vulnerable group to quantify their role in the incidence of the disease in the general population and evaluate environmental measures that affect tuberculosis incidence.

## Conclusions

In conclusion, this study estimates associations between socioeconomic variables and tuberculosis incidence. We also identified household crowding as a probable target for intervention to mitigate the impact of poverty and income inequality on the risk of tuberculosis. In addition, this study highlights the necessity to develop preventive interventions in prisons where vulnerable population become an independent determinant of the disease burden caused by tuberculosis. Finally, our results support the increase of primary care coverage as a strategy to control tuberculosis.
